# Modified Polyacrylic Acid-Zinc Composites: Synthesis, Characterization and Biological Activity

**DOI:** 10.3390/molecules21030292

**Published:** 2016-02-29

**Authors:** Mohammed Rafi Shaik, Mufsir Kuniyil, Mujeeb Khan, Naushad Ahmad, Abdulrahman Al-Warthan, Mohammed Rafiq H. Siddiqui, Syed Farooq Adil

**Affiliations:** Department of Chemistry, College of Science, King Saud University, P. O. Box 2455, Riyadh 11451, Saudi Arabia; rafiskm@gmail.com (M.R.S.); mufsir@gmail.com (M.K.); kmujeeb@ksu.edu.sa (M.K.); exactlykot@gmail.com (N.A.); awarthan@ksu.edu.sa (A.A.-W.); rafiqs@ksu.edu.sa (M.R.H.S.)

**Keywords:** polyacrylic acid, zinc, metal composite, biological activity

## Abstract

Polyacrylic acid (PAA) is an important industrial chemical, which has been extensively applied in various fields, including for several biomedical purposes. In this study, we report the synthesis and modification of this polymer with various phenol imides, such as succinimide, phthalimide and 1,8-naphthalimide. The as-synthesized derivatives were used to prepare polymer metal composites by the reaction with Zn^+2^. These composites were characterized by using various techniques, including NMR, FT-IR, TGA, SEM and DSC. The as-prepared PAA-based composites were further evaluated for their anti-microbial properties against various pathogens, which include both Gram-positive and Gram-negative bacteria and different fungal strains. The synthesized composites have displayed considerable biocidal properties, ranging from mild to moderate activities against different strains tested.

## 1. Introduction

The surge of interest in polymer science and engineering has been governed by the fact that there are multiple applications in various domains of daily life that can be explored for the new products formed. Improved performance and durability serve as a primary motive of the research. Among various polymers that are being studied, polyacrylic acid (PAA) has received significant attention due to its tremendous applications in several fields, including electrochemical [1], electronic [2], biomedical [3,4,5,6], and so on. It has been widely applied in various optical devices [7], as a solid electrolyte for lithium ion batteries, [8] in supercapacitors [9,10], as an ion exchanger [11], an efficient corrosion inhibitor [12] and eco-friendly coatings agent [13]. Furthermore, it has also been reported that the chemical stability of metal oxide nanoparticles in electronic and electrochemical devices could be improved by the use of PAA coatings [14]. Therefore, there is a growing interest in the development of PAA-based composites containing different oxide nanoparticles in a PAA matrix, such as PAA-TiO_2_ composites, *etc.* These types of composites have been extensively applied for various applications in solar cells [15], wastewater treatment [16], protective coatings [17], biomedical [18] and optical devices [19]. For instance, in a recent study, the pH-sensitive PAA-SiO_2_ composites were successfully used for the purpose of drug delivery [20]. Similarly, in other studies, it was revealed that the PAA is able to capture heavy metal ions at low concentrations, which can be used as a chelating material; hence, it can be utilized for the removal of toxic heavy metals from water [21].

Biomedical applications of various polymers and polymer-based materials, including polyacrylic acid (PAA) and its derivatives, have been extensively reported in the literature [22,23,24]. These materials are employed for the manufacturing of medical probes for analysis, hand-held water filters, surface coatings, fibrous disinfectants, and so on. Therefore, the biocidal property of the polymeric substance is also an important parameter, which has significant impact on various biological applications. For instance, Zauresh and coworkers carried out the synthesis of PAA complexes with streptomycin sulfate and the study of the antibacterial properties of PAA and their derivatives [25]. It was reported that the synthesized PAA complexes possessed improved antimicrobial property.

Gottenbos *et al.* [26] investigated the antimicrobial activity of poly(methacrylic acid) copolymers and described that electrostatic effects due to negatively-charged surfaces merely delay bacterial attachment and biofilm formation [26,27]. Other studies have revealed that polyacrylic acid based copolymers also showed excellent antimicrobial effects. For instance, in a recent report, it was revealed that cold plasma embedded acrylic acid on a poly(ethylene) surface produces a zone of inhibition for Staphylococcus aureus [28]. In another study, radiation embedded acrylic acid on poly(ethylene terephthalate) surfaces with 48 wt % acrylic acid decreased the integer of colony forming units (CFU) of Escherichia coli by 78% after 240 min [29]. Further, 40 wt % radiation embedded poly(methacrylic acid) on poly(propylene) surfaces decreased the progress of S. aureus and Klebsiella pneumoniae by above 99% after 24 h [30]. Similarly, Yang *et al.* [31] noticed that the antimicrobial activity effect against Pseudomonas aeruginosa rises with increasing mass fraction (from 10% to 40%) of radiation embedded polyacrylic acid on poly(propylene) nonwoven material added to a bacterial suspension [31]. However, in all of these works, the researchers concentrated on various antimicrobial agents and stated that the polyacrylic acid grafted carrier polymer was antimicrobially active also.

The prospect of integrating antimicrobial cross-linkers into polyacrylic acid castoff in such devices might be a technique of decreasing dermatitis and smell from the bacterially-arbitrated analysis of urine. Interpenetrated polymer networks (IPN) have displayed certain potential as a method of integrating antimicrobial agents into polymers, such as tetracycline [32,33], antibiotic streptomycin sulfate [25] or where the polymer network acts as a transporter for the antibiotics, for usage as drug delivery systems. Polymer networks have also been combined with numerous metals with antimicrobial properties, such as zinc and silver, to deliver antimicrobial hydrogels [34,35,36]. Zinc has been recognized in numerous biological methods, being accountable for regulatory and structural systems in both eukaryotic and prokaryotic cells. It possesses antifungal, antiviral and antibacterial properties [37,38].

Based on the above literature reports and owing to the growing interest in the development of composites in a PAA matrix, we herein report the synthesis of different PAA-metal based composites that are modified with various pendant phenyl imide groups. The as-prepared PAA-based composites were characterized using various methods, such as scanning electron microscopy (SEM), X-ray powder diffraction (XRD), Fourier-transform infrared (FT-IR) spectroscopy, differential scanning calorimetry (DSC) and thermal gravimetric analysis (TGA). Furthermore, these composites have been evaluated for their antimicrobial properties against various pathogens.

## 2. Results and Discussion

### 2.1. Scanning Electron Microscopy

**Figure 1:**
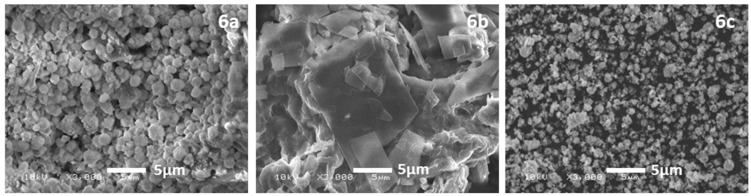
SEM micrographs of **6a**, **6b** and **6c**.

The surface morphology of the as-prepared PAA-based composites was further evaluated by scanning electron microscopy (SEM). The SEM images of different PAA-based composites are given in Figure 1, which were obtained at room temperature. It was observed that, morphologically, the composites possess well-defined and sharp edges; however, there is no consistency among the three composites tested. For example, Composite **6a** exhibited a tiny rock-like morphology, while **6b** appears to have a sheet/slab-like morphology with irregular stacking, whereas Composite **6c** has shown a spherical bead-like morphology with no consistency in size, which is in the range of 0.5 to 2 μm.

### 2.2. FT-IR Analysis

The formation of various PAA-metal-based composites was monitored by FT-IR analysis. For this purpose, the FT-IR spectra of intermediated compounds polyacrylic acid **2**, amino phenol derivative of polyacrylic acid **3** and imides-modified compounds (**5a** to **5c**) formed during the reaction, and the final composites were measured. For instance, the FT-IR spectrum of polyacrylic acid **2** consists of various peaks, which points towards the presence of the acrylic group, such as a peak located at 1710 cm^−1^ being attributed to the stretching vibration of the carbonyl group of carboxylic acid; the peak at 1402 cm^−1^ was attributed to the bending vibration of -C-H; and also, the occurrence of the peak at 1234 cm^−1^ was attributed the C-O of acid or ester; whereas, the appearance of the new peaks in the range of 3360 to 3700 cm^−1^ belonging to the amino phenol in the IR spectrum of amino phenol derivative of polyacrylic acid **3** confirms the transformation of polyacrylic acid **2** to amino phenol derivative of polyacrylic acid **3**. Apart from this, the IR spectrum of the amino phenol derivative of polyacrylic acid **3** also consists of some additional characteristics peaks, such as the peak at 2921 cm^−1^ was attributed the symmetrical stretching vibration of C-H and aromatic C-H, and the peak at 1736 cm^−1^ appears to belong to the carbonyl group of carboxylic acid. In the spectra, the peaks at 1612 cm^−1^, 1567 cm^−1^, 1507 cm^−1^ and 1458 cm^−1^ were attributed to the stretching vibrations of C=C. The peak at 1355 cm^−1^ was attributed to the vibration of -C-H- bending, and the peak at 1220 cm^−1^ was attributed to the stretching vibration of C-O of the acid or ester. All of these peaks confirm the presence of both the amino phenol group, as well as the PAA back bone in the compound.

**Figure 2:**
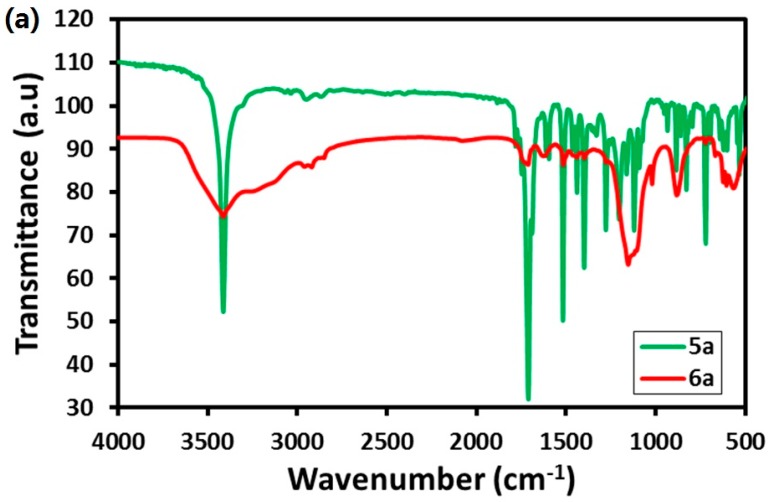

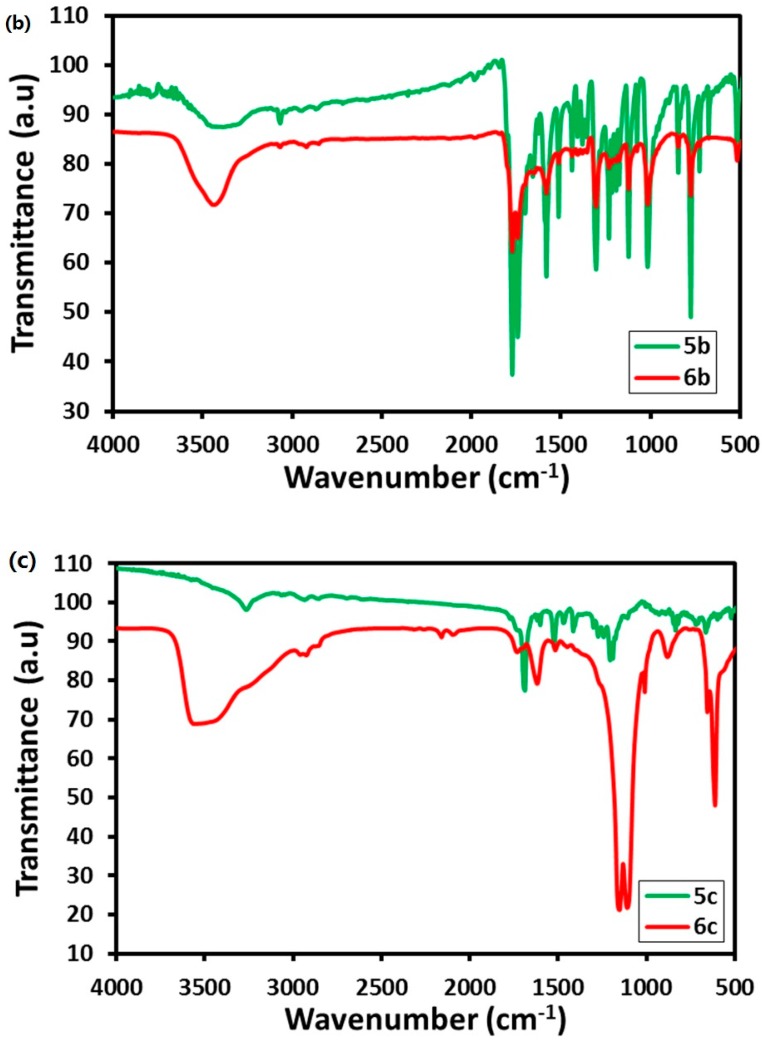
FT-IR spectra of **5a** and **6a** (**a**); **5b** and **6b** (**b**); **5c** and **6c** (**c**).

Furthermore, the FT-IR spectra of the imides-modified compounds (**5a** to **5c**) is compared to the FT-IR spectra of zinc complexes of the imide derivative of polyacrylic acid (**6a** to **6c**) as shown in Figure 2a–c to confirm the formation of the final composites. As per the literature, zinc is reported to produce a shift in the expected IR values by affecting the electron density in the bonded atoms and also has the capability to block out other prominent peaks [39]. Clearly, as shown in Figure 2a–c, some of the peaks are either missing from the spectra of final composites or they have slightly shifted from the original positions due to the addition of zinc. This confirms the presence of zinc in the final composites and also the formation of zinc complexes of the imide derivative of polyacrylic acid **6a** to **6c**. For instance, Figure 2a shows the IR spectra of **5a** and **6a**. In the IR spectrum of **6a**, the peaks at 528, 785, 933, 1071 and 1327 cm^−1^ are missing, whereas some other peaks are slightly shifted, such as the peak at 1746 cm^−1^ belonging to the C=O group; 1120 cm^−1^ related to the N-C band stretching in **5a** is slightly shifted to 1740 cm^−1^ and 1152 cm^−1^, respectively, in the IR spectrum of **6a**. Similar kinds of shifts are also observed in the IR spectra of **6b** and **6c**, as shown in Figure 2b,c, respectively. The detail information of other prominent IR peaks of the Compounds **5a** to **5c** and **6a** to **6c** are given below.

#### 2.2.1. Imide Derivative of Polyacrylic Acids **5a** to **5c**

**5a**: ν 3068 cm^−1^ (C-H aromatic and C-H stretching), 1748 cm^−1^ (C=O carboxylic acid), 1690 cm^−1^ (C=C), 1611 cm^−1^ (C=C), 1595 cm^−1^ (C=C), 1518 cm^−1^ (C=C), 1399 cm^−1^ (-C-H- bending), 1278 cm^−1^ (C-O acid or ester), 1204 cm^−1^ (C-O acid or ester), 1120 cm^−1^ (N-C).

**5b**: ν 3066 cm^−1^ (C-H aromatic and C-H stretching), 1740 cm^−1^ (C=O carboxylic acid), 1699 cm^−1^ (C=C), 1589 cm^−1^ (C=C), 1580 cm^−1^ (C=C), 1512 cm^−1^ (C=C), 1437 cm^−1^ (C=C), 1303 cm^−1^ (-C-H bending), 1231 cm^−1^ (C-O acid or ester), 1121 cm^−1^ (N-C).

**5c**: ν 3064 cm^−1^ (C-H aromatic and C-H stretching), 1769 cm^−1^ (C=O carboxylic acid), 1687 cm^−1^ (C=C), 1600 cm^−1^ (C=C), 1522 cm^−1^ (C=C), 1469 cm^−1^ (C=C), 1413 cm^−1^ (-C-H bending), 1241 cm^−1^ (C-O acid or ester), 1205 cm^−1^ (C-O acid or ester), 1190 cm^−1^ (N-C).

#### 2.2.2. Zinc Complexes of the Imide Derivative of Polyacrylic Acid **6a** to **6c**

**6a**: ν 2956 cm^−1^ (C-H aromatic and C-H stretching), 2866 cm^−1^ (C-H aromatic and C-H stretching), 1591 cm^−1^ (C=C), 1516 cm^−1^ (C=C), 1439 cm^−1^ (C=C), 1398 cm^−1^ (-C-H- bending), 1277 cm^−1^ (C-O acid or ester), 1152 cm^−1^ (N-C).

**6b**: ν 1740 cm^−1^ (C=O), 1580 cm^−1^ (C=C), 1511 cm^−1^ (C=C), 1437 cm^−1^ (C=C), 1407 cm^−1^ (-C-H- bending), 1353 cm^−1^ (-C-H bending), 1303 cm^−1^ (-C-H- bending), 1231 (C-O acid or ester), 1214 (C-O acid or ester), 1190 cm^−1^ (N-C), 1075 cm^−1^ (N-C), 1017 cm^−1^ (N-C).

**6c**: ν 1736 cm^−1^ (C=O), 1507 cm^−1^ (C=C), 1153 cm^−1^ (N-C), 1020 cm^−1^ (N-C).

### 2.3. ^1^H-NMR Analysis

**Figure 3:**
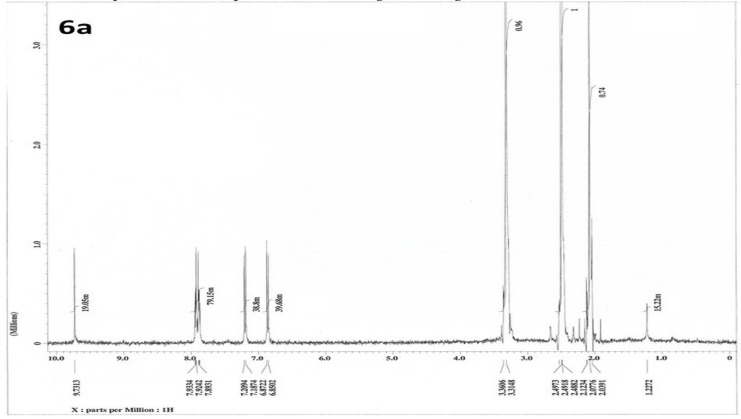

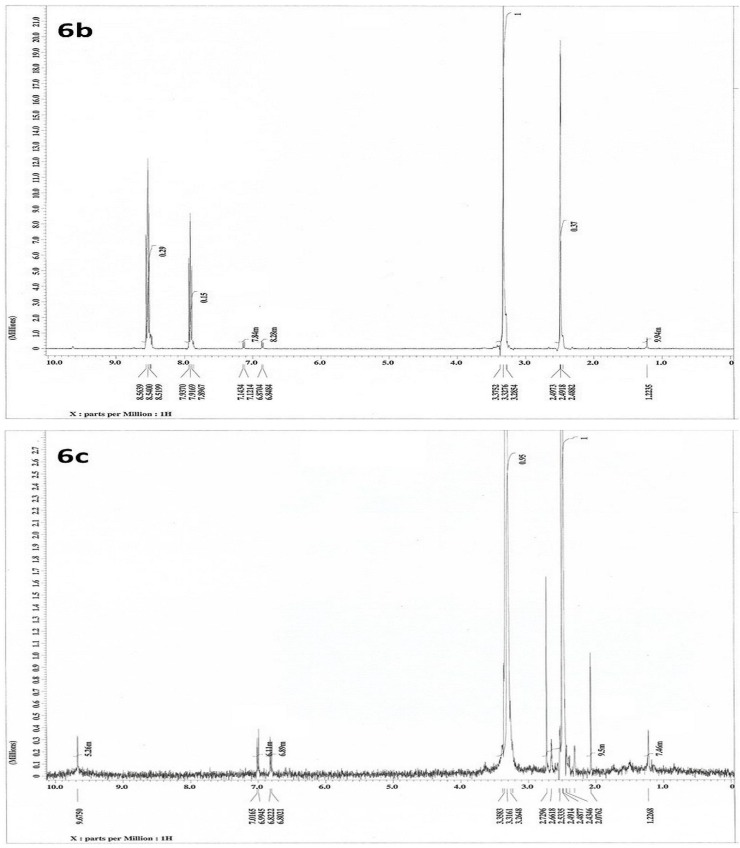
^1^H-NMR spectrum of **6a**, **6b** and **6c**.

Additionally, the formation of zinc complexes of the imide derivative of polyacrylic acid from **6a** to **6c** is also confirmed by proton NMR, as shown in Figure 3, respectively. The ^1^H-NMR spectrum has given information about the presence of the polymeric back bone and the derivatization of the polymer along with the imides. The presence of the signals are in the aromatic region of the NMR δ ppm 6.5 to 8. The presence of signals at the δ ppm 9.6 to 9.8 may be due to the presence of carboxylic acid groups on the polymeric chain, which could be due to incomplete derivatization. The detailed information of other prominent NMR signals of **5a** to **5c** and Composites **6a** to **6c** are given below. The ^1^H-NMR spectrum of Composites **6a** to **6c** are given in Figure 3.

Polyacrylic acid **2**: ^1^H-NMR (400 MHz, DMSO-*d*_6_): δ ppm 3.56 (s, 10H), 7.09 (s, 1H).

Amino phenol derivative of polyacrylic acid **3**: ^1^H-NMR (400 MHz, DMSO-*d*_6_): δ ppm 1.44–1.55 (m, 2H), 3.36 (t, 9H), 6.61–6.81 (dd, 12H), 7.90 (s, 1H), 9.12 (s, 2H).

#### 2.3.1. Imide Derivative of Polyacrylic Acids **5a** to **5c**

**5a**: ^1^H-NMR (400 MHz, DMSO-*d*_6_): δ ppm 3.34 (s, 4H), 6.87 (d, 2H), 7.21(d, 2H), 7.82 to 8.00 (m, 4H), 9.74 (s, 1H).

**5b**:^1^H-NMR (400 MHz, DMSO-*d*_6_): δ ppm 2.09 (s, 1H), 3.35 (s, 18H), 7.93 (s, 2H), 8.54 (s, 4H).

**5c**:^1^H-NMR (400 MHz, DMSO-*d*_6_): δ ppm 1.48 to 1.66 (m, 1H), 1.7 to 1.8 (m, 1H), 2.74 (s, 4H), 3.26 to 3.44 (m, 8H), 3.65 (t, 1H), 6.82 (d, 2H), 7.01 (d, 2H), 9.69 (s, 1H).

#### 2.3.2. Zinc Complexes of the Imide Derivative of Polyacrylic Acid **6a** to **6c**

**6a**: ^1^H-NMR (400 MHz, DMSO-*d*_6_): δ ppm 1.22 (s, 1H), 6.85–6.87 (d, 2H), 7.18–7.20 (d, 2H), 7.89 to 7.93 (m, 5H), 9.73 (s, 1H).

**6b**: ^1^H-NMR (400 MHz, DMSO-*d*_6_): δ ppm 1.22 (s, 1H), 6.84 to 6.87 (d, 1H), 7.12 to 7.14 (d, 1H), 7.89 to 7.93 (m, 20H), 8.51 to 8.56 (m, 40H).

**6c**: ^1^H-NMR (400 MHz, DMSO- *d*_6_): δ ppm 1.22 (s, 1H), 2.07 (s, 2H), 6.80–6.82 (d, 1H), 6.99–7.01 (d, 1H), 9.67 (s, 1H).

### 2.4. Thermo-Gravimetric Analysis

**Figure 4:**
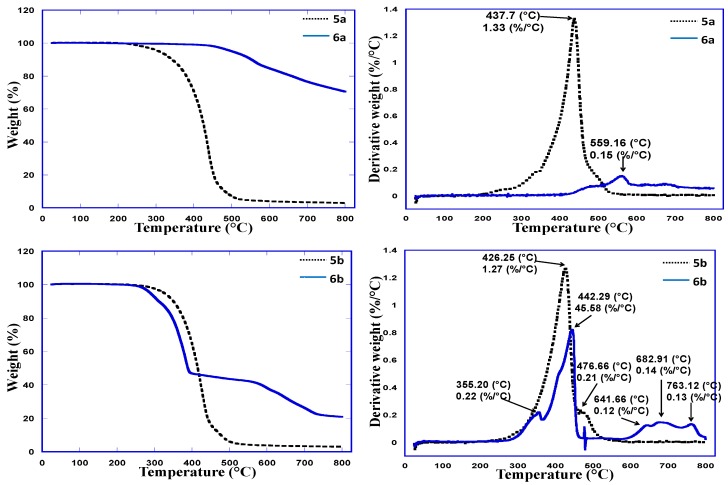

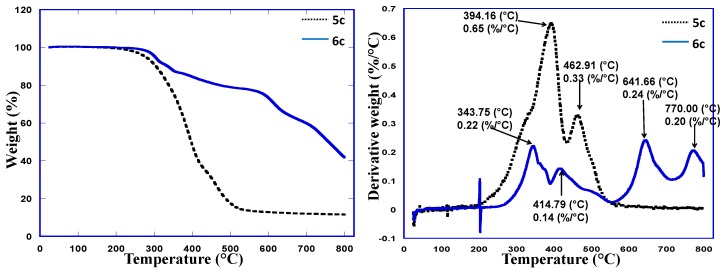
TGA graphs of **5a** and **6a**, **5b** and **6b** and **5c** and **6c**.

TGA results for the polymers and their metal composite systems prepared in this study are shown in Figure 4. The polymers and their metal composites were subjected to heating up to 800 °C at the rate of 25 °C/min. The results were compared, and it was observed that the metal composites were thermally more stable than their precursor. The weight loss at different temperatures is tabulated in Table 1.

### 2.5. Differential Scanning Calorimetry

**Figure 5:**
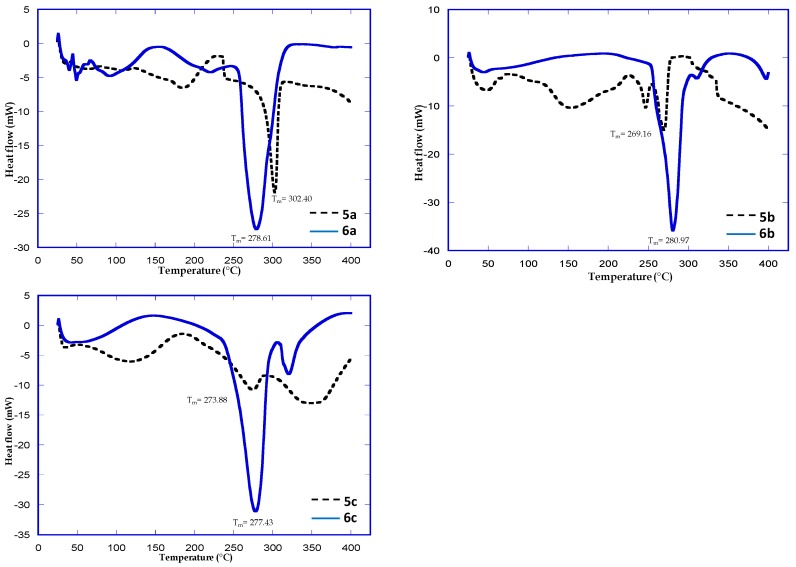
DSC spectra of polymer Compound **5a** and **6a**, **5b** and **6b** and **5c** and **6c**.

DSC results for the polymers and their metal composite systems prepared in this study are shown in Figure 5. In order to further investigate the influence of Zn(II) complexation on the thermal property of the polymer, the differential scanning calorimetry studies were carried out for the polymer and the Zn(II) complex. From the thermogram of pure polymer, the melting point (Tm) of **5a** to **5c** was found at 302.4 °C, 269.16 °C and 273.88 °C, respectively, while the metal complexes of the polymers **6a** to **6c**, the melting point (Tm) was found to be 278.61 °C, 280.97 °C and 277.43 °C, respectively. This shift could be due to the interaction that occurs between the hydrophilic part of polymer and the Zn(II) metal ion and also gives an idea of the degree of complexation with the metal ion, which was found to be different for different polymers.

### 2.6. Biological Evaluation

#### 2.6.1. Antibacterial Activity

The *in vitro* antibacterial activity of the newly-synthesized **6a** to **6c** (Shown in Table 2) has been studied against the bacterial strains *Staphylococcus aureus* (MTCC 96), *Klebsiella planticola* (MTCC 530), *Bacillus subtilis* (MTCC 121), *S. aureus MLS16* (MTCC 2940), *Micrococcus luteus* (MTCC 2470), *Escherichia coli* (MTCC 739) and *Pseudomonas aeruginosa* (MTCC 2453) by the agar well diffusion method [40,41,42], and the strains were obtained from the Institute of Microbial Technology (IMTECH), Chandigarh. Neomycin was used as a standard drug.

The MIC values obtained from the following experiments were compared to neomycin as a standard, and it was observed that the Compounds **6b** and **6c** were found to be selectively active against *Staphylococcus aureus* MTCC 96 with biocidal activity equivalent to the standard used. The compounds have shown mild to very mild activity against the rest of the bacterial strains against which they were tested.

#### 2.6.2. Antifungal Activity

The *in vitro* antifungal activity of the newly-synthesized **6a** to **6c** (Shown in Table 3)has been studied against the fungal strains *Candida albicans* (MTCC 227), *Candida albicans* (MTCC 854), *Candida albicans* (MTCC 1637), *Candida albicans* (MTCC 3017), *Candida albicans* (MTCC 3018), *Candida albicans* (MTCC 3958), *Candida albicans* (MTCC 4748), *Candida albicans* (MTCC 7315), *Candida aaseri* (MTCC 1962), *Candida glabrata* (MTCC 3019), *Candida parapsilosis* (MTCC 1744), *Issatchenkia hanoiensis* (MTCC 4755) and *Issatchenkia orientalis* (MTCC 3020) by the agar well diffusion method [40,41,42], and the strains were obtained from the Institute of Microbial Technology (IMTECH), Chandigarh. Fluconazole was used as a standard drug.

The MIC values obtained from the following experiments were compared to the fluconazole as a standard, and it was observed that the newly-synthesized compounds have exhibited mild to very mild activity against the fungal strains tested. The synthesized derivatives exhibited activity against fungal strain *Issatchenkia orientalis* comparable to the standard used.

## 3. Experimental Section

### 3.1. Materials and Strains

Acrylic acid was purchased from Alpha Chemika, Mumbai, India, while ammonium peroxodisulfate and glacial acetic acid were procured from Qualikems, New Delhi, India. 4-amino phenol, succinic anhydride and thionyl chloride were purchased from BDH Chemicals Limited, Bristol, England. Zinc sulfate and phthalic anhydride were procured from Riedel-de Haen, Hanover, Germany, while cupric nitrate and naphthalic anhydride were from Fluka, Buchs, Switzerland. Solvents toluene, methanol and tetrahydrofuran were purchased from Panreac, Barcelona, Spain, and Merck, Darmstadt, Germany, respectively. All of the solvents were used as such without further purification.

### 3.2. Methods

#### 3.2.1. Synthesis of Polyacrylic Acid **2**

Polymerization of acrylic acid was carried out using the procedure reported earlier [43] in which ammonium peroxodisulfate was used to catalyze the reaction. Acrylic acid and ammonium peroxodisulfate were taken in 2:3 molar ratios. Ten milliliters (0.145 moles) of acrylic acid were dissolved in the least amount of toluene treated with aqueous solution of 41.5 g (0.2175 moles) of ammonium peroxodisulfate at 60 °C for 6 h. The pale yellow solid product obtained was isolated by vacuum filtration of the reaction mixture using a Buchner funnel. The precipitate was washed thoroughly with water to get rid of any excess catalyst or unreacted monomer that may be present in the precipitate formed. The pale yellow solid product obtained was dried in a vacuum oven at 70 °C. The product was confirmed by ^1^H-NMR studies.

#### 3.2.2. Synthesis of Amino Phenol Derivative of Polyacrylic Acid **3**

**Figure 6:**
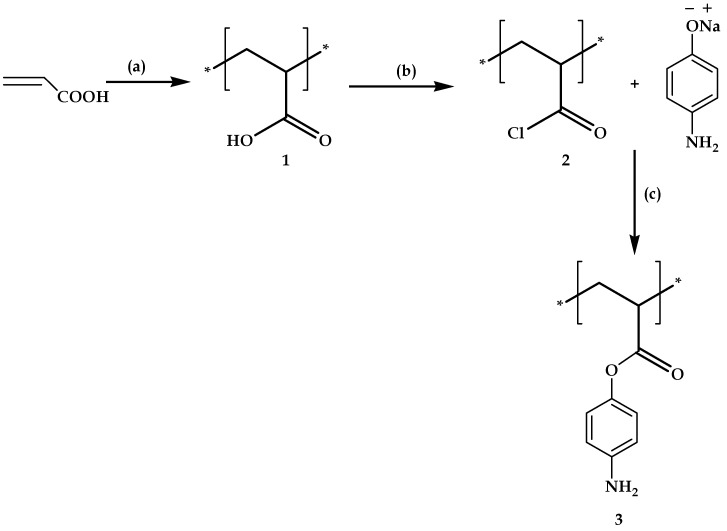
Synthesis of amino phenol derivative of polyacrylic acid **3**. *Reagents and conditions:* (a) APS, toluene, 60 °C, 6 h; (b) SOCl_2_, N_2_, 48 h; (c) aqueous NaOH, 4-aminophenol, 30 min.

Polyacrylic acid was converted to corresponding acid chloride followed by coupling with 4-amino phenoxide (shown in Figure 6). Ten grams of polyacrylic acid were taken in a round bottomed flask, to which equimolar thionyl chloride was added, and the resulting solution was stirred for 48 h at room temperature in an inert atmosphere. This yielded the acid chloride derivative of the polyacrylic acid, which was further treated with sodium phenoxide, which was prepared by dissolving 23.9 g of 4-amino phenol in a 1.5 M solution of 8.8 g of sodium hydroxide and was stirred at room temperature for 30 min. The black colored solid formed was filtered using the vacuum filtration process. This was washed several times with water and dilute HCl. The obtained solid was dried in a vacuum oven at 70 °C. The product was confirmed by ^1^H-NMR studies.

#### 3.2.3. Synthesis of Imide Derivative of Polyacrylic Acids **5a** to **5c**

**Figure 7:**
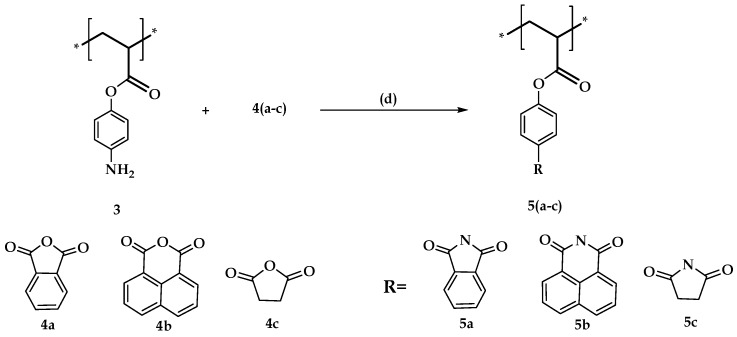
Synthesis of imide derivative of polyacrylic acids **5a** to **5c**. *Reagents and conditions:* (d) acetic acid, 130 °C, reflux, 5 h.

Imide Derivative of Polyacrylic Acids **5a** to **5c** is shown in Figure 7. Ten grams of **3** and equimolar anhydride were taken in a round bottom flask fitted with reflux condenser. In 100 mL acetic acid medium, the reaction mixture was refluxed for 5 h. The black-colored solid formed was filtered using a vacuum filtration process, which was washed several times with water. The obtained solid was dried in a vacuum oven at 70 °C. The product was confirmed by ^1^H-NMR studies.

#### 3.2.4. Synthesis of Zinc Complexes of the Imide Derivative of Polyacrylic Acid **6a** to **6c**

**Figure 8:**
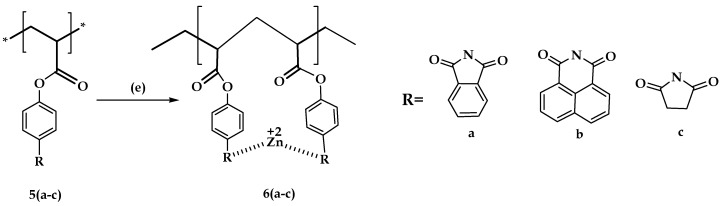
Synthesis of zinc complexes of the imide derivative of polyacrylic acid **6a** to **6c**. *Reagents and conditions:* (e) Zinc sulfate heptahydrate, CH_3_OH, THF, 20 min.

The zinc composite of the desired structure was made using zinc sulfate heptahydrate (shown in Figure 8). Five hundred milligrams of the amide derivative of PAA, dissolved in 5 mL of tetrahydrofuran, were mixed with an equimolar quantity of zinc salt solution, which was made by dissolving 3 mL of methanol, with constant stirring for 20 min. The product obtained was separated using vacuum filtration using a Buchner funnel, washed several times with distilled water and dried at 70 °C. The yield is 262 mg.

### 3.3. Methods of Characterization

Powder X-ray diffraction (XRD) studies were carried out using an Altima IV Regaku X-ray diffractometer. Fourier transform infrared spectra (FT-IR) were recorded on a Perkin-Elmer spectrum 1000 FT-IR spectrophotometer. ^1^H-NMR spectra were obtained on an automated JEOL Eclipse-400 spectrometer in dimethyl sulphoxide-*d*_6_. Thermal gravimetric analysis (TGA) was measured on a Mettler Toledo TGA1 and differential scanning calorimetry (DSC) was measured on a Mettler Toledo DSC1. TGA was done in the temperature range of 25 to 800 °C in nitrogen gas, whereas DSC a range of 25 to 400 °C. Approximately 7 mg of sample were taken in this study.

### 3.4. Method of Antimicrobial Activity

#### Bacterial and Fungal Strains

For determining the antimicrobial activity, the following strains of bacteria and fungi were used. All of the strains were obtained from the Microbial Type Culture Collection (MTCC). *Staphylococcus aureus* (MTCC 96), *Klebsiella planticola* (MTCC 530), *Bacillus subtilis* (MTCC 121), *S. aureus MLS16* (MTCC 2940), *Micrococcus luteus* (MTCC 2470), *Escherichia coli* (MTCC 739), *Pseudomonas aeruginosa* (MTCC 2453). *Candida albicans* (MTCC 227), *Candida albicans* (MTCC 854), *Candida albicans* (MTCC 1637), *Candida albicans* (MTCC 3017), *Candida albicans* (MTCC 3018), *Candida albicans* (MTCC 3958), *Candida albicans* (MTCC 4748), *Candida albicans* (MTCC 7315), *Candida aaseri* (MTCC 1962), *Candida glabrata* (MTCC 3019), *Candida parapsilosis* (MTCC 1744), *Issatchenkia hanoiensis* (MTCC 4755), *Issatchenkia orientalis* (MTCC 3020).

For growing the strains, the recommended medium was used and for long-term preservation. Strains were stored at −80 °C in 20% glycerol.

Antibacterial activity of the composites was tested using the standard agar diffusion method [40,41,42]. Cultures of bacteria were grown to log phase in their respective recommended medium. These cultures were mixed with sterile soft agar, and the mixture was subsequently poured on the surface of respective agar plates. Wells were created in the agar using a sterile borer. Finally, the materials to be tested were added to the wells, and plates were incubated at temperatures recommended for bacteria for optimal growth. The zone of inhibition was measured using a scale and was recorded after 2 to 3 days of incubation.

The antifungal activity against various species of Candida was also determined by using the standard agar diffusion method, as described above, except that the Sabouraud dextrose agar medium was used.

## 4. Conclusion

This work explores the new derivatives of polyacrylic acid, a well-known polymer, designed and synthesized with the aim to improve and enhance its properties. The synthesized derivatives were found to possess a varied degree of complexation with the metal salt based on the DSC results and better thermal stability on the TGA analysis. The biological evaluation revealed that the displayed zinc derivative of one polymer was found to possess excellent activity against *Staphylococcus aureus* bacterial strain, while moderate to mild activity against the bacterial strains tested, while it displayed mild activity against the fungal strains tested.

## Figures and Tables

**Table 1:** Weight loss percentage for the polymers Compounds **5a** to **5c** and **6a** to **6c**.

Sample	Weight Loss % at Various Temperatures						
	200 °C	300 °C	400 °C	500 °C	600 °C	700 °C	800 °C
**5a**	0	4.16	29.48	92.6	96.1	96.49	96.89
**6a**	0	0.26	1.04	4.94	15.45	23.64	29.49
**5b**	0	2.6	35.71	93.77	96.1	96.49	96.88
**6b**	0	7.66	53.25	56.36	61.82	73.51	78.96
**5c**	0.26	8.84	52.08	82.47	87.14	87.92	88.31
**6c**	0	4.55	15.45	20.91	26.75	40.39	58.31

**Table 2:** Antibacterial activity of zinc complexes of modified polyacrylic acid **6a**, **6b** and **6c**.

Test Compounds ^a^	MIC ^b^ (μg/mL)			
	6a	6b	6c	Neomycin
*Staphylococcus aureus* MTCC 96	300	18.75	18.75	18.75
*Klebsiella planticola* MTCC 530	300	75	150	18.75
*Bacillus subtilis* MTCC 121	75	75	37.5	18.75
*S. aureus MLS16* MTCC 2940	150	150	150	18.75
*Micrococcus luteus* MTCC 2470	150	150	37.5	18.75
*Escherichia coli* MTCC 739	150	150	75	18.75
*Pseudomonas aeruginosa* MTCC 2453	150	75	75	18.75

^a^ MTCC = Microbial Type Culture Collection, Institute of Microbial Technology, Chandigarh, India; ^b^ MIC = minimum inhibitory concentration.

**Table 3:** Antifungal activity of zinc complexes of modified polyacrylic acid **6a**, **6b** and **6c**.

Test Compounds	Minimum Inhibitory Concentration (μg/mL)			
	6a	6b	6c	Fluconazole
*Candida albicans* MTCC 183	75	75	75	32
*Candida albicans* MTCC 227	75	75	75	32
*Candida albicans* MTCC 854	75	75	75	32
*Candida albicans* MTCC 1637	75	75	75	64
*Candida albicans* MTCC 3017	75	75	75	64
*Candida albicans* MTCC 3018	150	150	150	32
*Candida albicans* MTCC 3958	75	75	75	64
*Candida albicans* MTCC 4748	75	75	75	32
*Candida albicans* MTCC 7315	75	75	75	32
*Candida aaseri* MTCC 1962	150	150	150	64
*Candida glabrata* MTCC 3019	150	150	150	64
*Candida parapsilosis* MTCC 1744	150	150	150	16
*Issatchenkia hanoiensis* MTCC 4755	300	300	300	128
*Issatchenkia orientalis* MTCC 3020	150	150	150	128
